# Experiment and Analysis of Variance for Stabilizing Fine-Grained Soils with Cement and Sawdust Ash as Liner Materials

**DOI:** 10.3390/ma17102397

**Published:** 2024-05-16

**Authors:** Sadiq Iliyas, Ahmad Idris, Ibrahim Haruna Umar, Hang Lin, Ahmad Muhammad, Linglin Xie

**Affiliations:** 1 School of Resources and Safety Engineering, Central South University, Changsha 410083, China; sadiqiliyasu11@gmail.com (S.I.); ibrahimharunaumar@yahoo.com (I.H.U.); 2 Department of Agriculture Engineering, Federal College of Land Resources Technology, Owerri 1518, Nigeria; 3 Department of Civil Engineering, Bayero University, Kano 3011, Nigeria; aidris.civ@buk.edu.ng; 4 Department of Civil Engineering, Kano State Polytechnic, Kano 3405, Nigeria; ahmad.civilengr@gmail.com; 5 Key Laboratory of Natural Resources Monitoring and Supervision in Southern Hilly Region, Ministry of Natural Resources, Changsha 430071, China; 6 Department of Investigation and Monitoring, The Second Surveying and Mapping Institute of Hunan Province, Changsha 430071, China

**Keywords:** stabilization, fine-grained soils, cement, sawdust ash (SDA), analysis of variance (ANOVA)

## Abstract

Due to volume change and low strength, fine-grained soils are problematic in construction. Stabilization with cement and sawdust ash (SDA) by-products can improve engineering properties. This study aimed to investigate the effectiveness of cement and sawdust ash (SDA) in stabilizing fine-grained soils for liner applications. Varying proportions of cement (0–9%) and SDA (0–10%) were added to soil samples (*n* = 24). Specimens were tested for unconfined compressive strength (UCS), hydraulic conductivity (HC), and volumetric shrinkage strain (VSS). Two-way ANOVA analyzed stabilization effects. Optimal stabilization occurred with 6% cement and 6% SDA, resulting in significant increases in UCS (51 to 375 kN/m^2^) and decreases in HC (1.7 × 10^−8^ to 4.7 × 10^−10^ m/s) and VSS (12.8 to 3.51%) compared to untreated soil. ANOVA indicated that both cement and SDA had statistically significant (*p* < 0.05) effects on improving all three engineering properties. The addition of 6% cement and 6% SDA significantly improved the expansive soil’s strength, hydraulic conductivity, and volume change properties. ANOVA confirmed the quantitative improvements and the significance of both stabilizers. Stabilization using the by-product SDA has the potential to be a sustainable soil improvement method.

## 1. Introduction

Clay liners are widely used in engineering applications such as landfill, waste containment, and groundwater protection. They act as barriers to prevent the migration of contaminants from the waste to the surrounding environment [1,2,3,4]. However, clay liners are susceptible to cracking and shrinking due to moisture content and temperature changes, which can compromise their performance and durability [5,6,7]. Therefore, improving the geotechnical properties of clay liners, such as strength, permeability, and shrinkage, is necessary to enhance their effectiveness and reliability [8,9,10]. One of the methods to improve the quality of clay liners is to stabilize them with additives, such as cement and sawdust ash (SDA) [11,12]. Cement is a common stabilizing agent that can increase the strength and reduce the permeability of clayey soils. However, cement also increases the brittleness and shrinkage of clay liners, which can lead to cracking and failure [13,14,15]. SDA is a waste material obtained from the combustion of sawdust, which is rich in silica and alumina. SDA can act as a pozzolanic material that reacts with cement to form additional cementitious products, improving the strength and durability of clay liners [16,17]. Moreover, SDA can reduce the shrinkage and cracking of clay liners by filling the pores and creating a more uniform structure [7]. SDA is also a low-cost and eco-friendly material that can utilize abundant and renewable sawdust resources and reduce the environmental impact of cement production [18].

There are still some research gaps and challenges in the use of cement and SDA for stabilizing fine-grained soils as clay liner materials, particularly in the context of unconfined compressive strength (UCS), hydraulic conductivity (HC), and volumetric shrinkage strain (VSS). Some research gaps are: the optimal proportion and dosage of cement and SDA for different fine-grained soils and clay liners [19]; the effect of curing time and temperature on the geotechnical properties and microstructure of cement and SDA-stabilized clay liners [20]; cement and SDA-stabilized clay liners’ long-term performance and durability under various environmental and loading conditions, such as wetting–drying cycles, freezing–thawing cycles, and chemical attacks [21,22]; the comparison and evaluation of cement and SDA-stabilized clay liners with other types of clay liners, such as geosynthetic clay liners, bentonite-enhanced sand liners, and polymer-modified clay liners [23], and; the economic and environmental feasibility and benefits of cement and SDA-stabilized clay liners in engineering projects [24].

Previous research has shown that the amendment of cement stabilization with sawdust ash has led to significant strength benefits in expansive and soft clayey soils [12]. Studies have also focused on the environmental impact of using sawdust ash as a stabilizer for expansive soils, emphasizing the need for soil stabilization due to high plasticity [25]. Analysis of variance (ANOVA) is commonly used in geotechnical research, especially in studies evaluating and comparing different soil properties and liner materials. When testing soil samples under various conditions, ANOVA helps researchers to determine whether differences between group means are statistically significant or simply due to chance. Several studies have used ANOVA to compare the properties of natural and compacted clay liners for landfill and containment facilities. Benson and Trast performed a one-way ANOVA on the hydraulic conductivity values of three clay liners compacted to different moisture contents and densities. The analysis showed significant differences between liner types and preparation methods [26]. Daniel et al. used ANOVA to compare consolidation parameters, permeability, and shear strength of compacted kaolinite and bentonite liners with natural clays. Their ANOVA results indicated that the engineered clays had superior containment capacities. As a result, ANOVA can be used to analyze the factors affecting clay liners’ integrity and performance. The technique confirms whether different additives, compositions, moisture levels, or compaction methods have a statistically significant effect on permeability, swelling potential, compressibility, shrinkage, and other properties that affect the effectiveness of clay liners for containment structures [27]. ANOVA provides quantitative validation of factors and trends affecting liner behavior that may not be apparent from the data alone [28].

**Figure 1:**
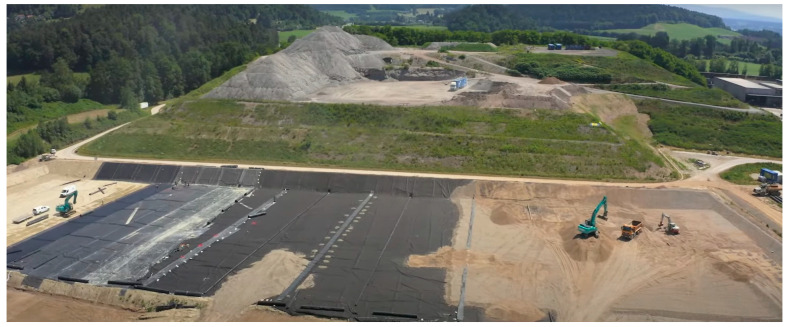
A liner system.

This study investigates the effectiveness of cement and SDA combination for stabilizing fine-grained soils as clay liner materials. The experimental design consists of preparing clayey soil samples with different proportions of cement and SDA and testing them for unconfined compressive strength (UCS), hydraulic conductivity (HC), and volumetric shrinkage strain (VSS). The analysis of variance (ANOVA) method is used to evaluate the significance of the effects of cement and SDA on the geotechnical properties of clay liners. The results of this study can provide valuable insights into the optimal design and application of cement and SDA-stabilized clay liners in engineering projects. Figure 1 shows a liner system.

## 2. Materials and Method

### 2.1. Materials

Table 1 presents the protocol for preparing soil samples to study the effectiveness of combining cement and sawdust ash (SDA) for stabilizing fine-grained soils as clay liner materials. The table includes SDA content (ranging from 0% to 10%), cement content (ranging from 0% to 9%), and compaction water content (−2%, 0%, +2%, and +4% of the optimum moisture content (OMC)). These variables were varied to produce different soil specimens that were then tested for unconfined compressive strength (UCS), hydraulic conductivity (HC), and volumetric shrinkage strain (VSS). The results of these tests were used to evaluate the effectiveness of the cement and SDA combination in stabilizing fine-grained soils. Initially, soil specimens were prepared by thoroughly mixing the fine-grained soil with predetermined percentages of cement (ranging from 0% to 9%) and SDA (ranging from 0% to 10%). Each mixture needed to be homogenized to ensure consistent distribution of the stabilizing agents throughout the soil. Water was then added to achieve the desired moisture content (−2%, 0%, +2%, and +4% of the OMC). The thoroughly mixed material was compacted into molds to form specimens, which follows the standard procedure for soil compaction, using a mold and hammer at controlled weights and heights to achieve specified density and moisture levels.

#### 2.1.1. Natural Soil

**Figure 2:**
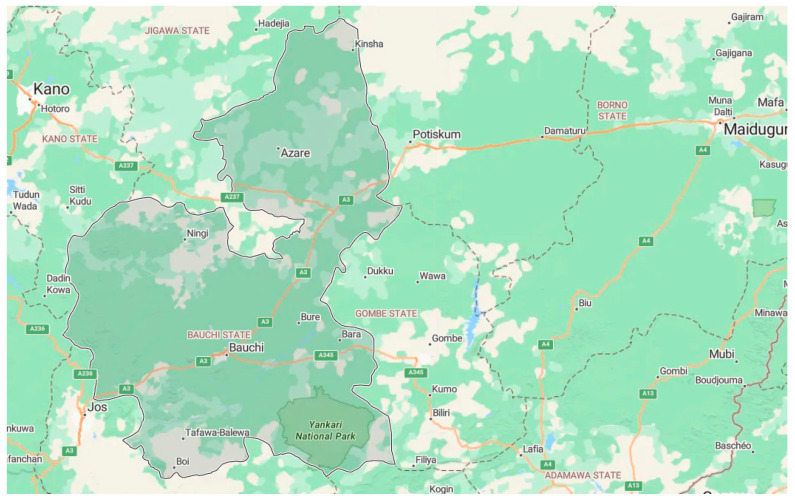
Bauchi-Gombe Road in Inkil Town location.

**Figure 3:**
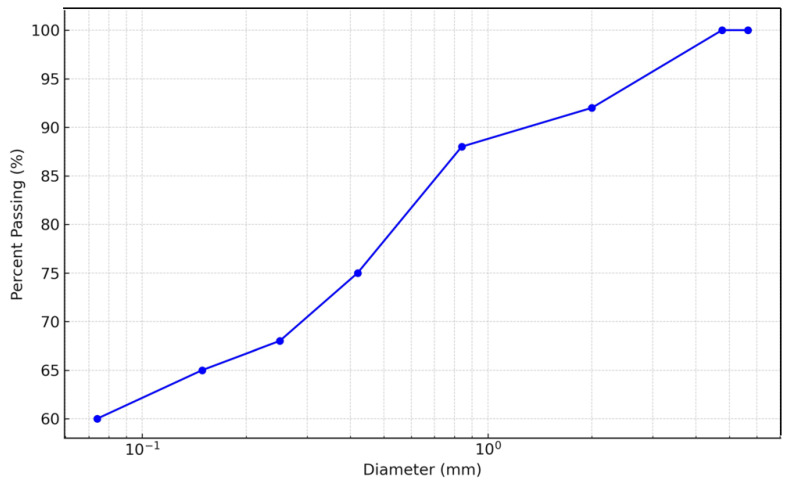
Particle size distribution curve.

**Figure 4:**
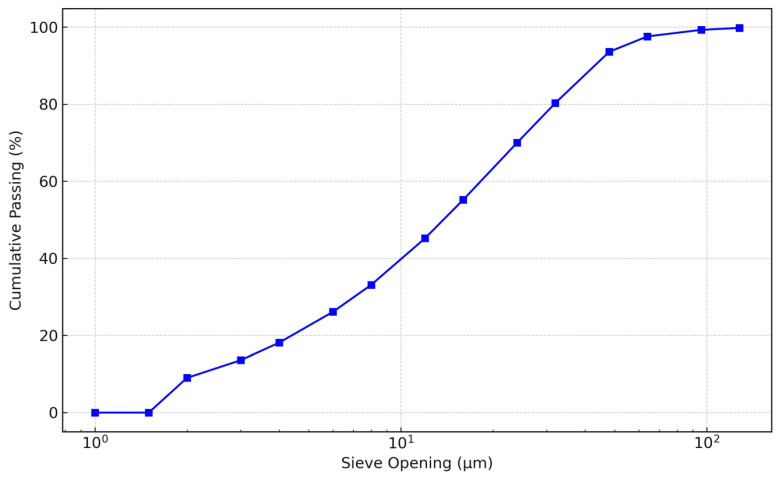
Particle size distribution curve of the cement.

**Figure 5:**
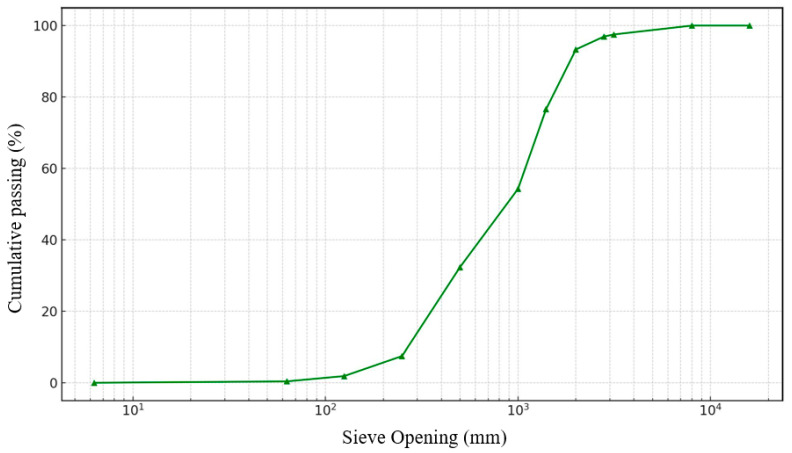
Particle size distribution curve of the sawdust ash.

Fine-grained soil specimens were obtained from a burrow area situated on the Bauchi-Gombe Road in Inkil town, within the Bauchi Local Government District (positioned at 10°18′40″ N, 9°53′46″ E) of Bauchi state, Nigeria (Figure 2 shows the location on a map). The sampling was performed utilizing the disrupted sampling method at a depth range of 0.5 to 1.0 m. The samples were gathered into medium-to-large bags, sealed, and transported to the lab for testing. The fundamental attributes of the untreated soil specimen, cement, and sawdust ash are outlined in Table 2, Table 3 and Table 4. The particle size distribution graph of the natural soil, cement, and sawdust ash are displayed in Figure 3, Figure 4 and Figure 5. Per the Unified Soil Classification System structure, the soil is categorized as CH.

#### 2.1.2. Sawdust Ash

**Figure 6:**
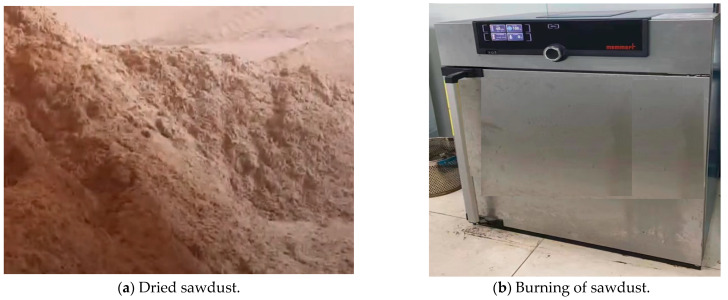

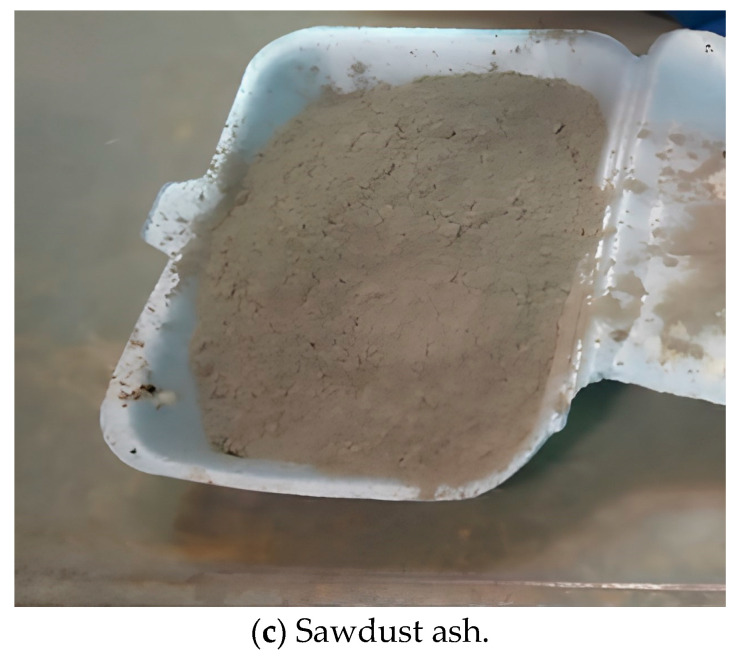
Sawdust process to ash.

The sawdust used in the study was sourced from a local sawmill in the Gwamaja Yan Katako Dala Industrial District in Kano State (see Figure 6a). Subsequently, the sawdust was cleaned to eliminate excessive bark and organic matter. It was then air-dried and incinerated in a combustion furnace at 500°C (see Figure 6b). The incinerated sawdust was stored in an air-tight container to avoid moisture exposure or contamination. The incinerated sawdust used in this study mainly comprised silica, alumina, and lime, with smaller amounts of other metal oxides, as shown in Table 3.

#### 2.1.3. Cement 

**Figure 7:**
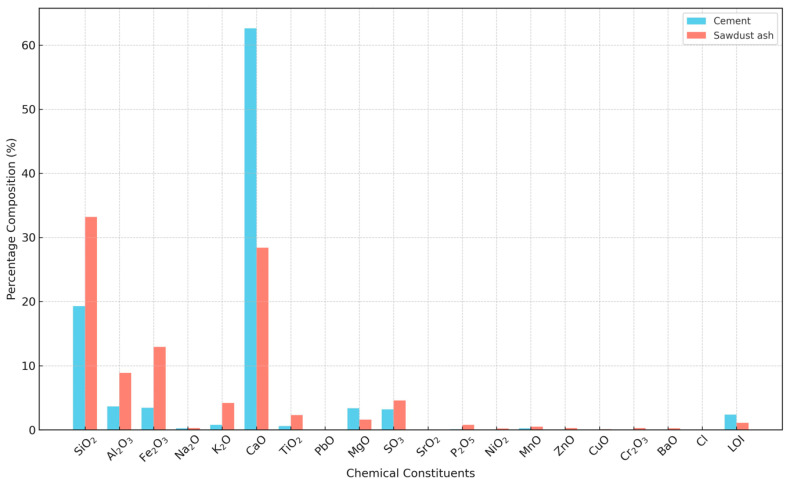
Percentage of oxide composition of cement and sawdust ash.

The ordinary Portland cement (OPC) utilized in this study was commercially obtained from a local market in Sharada, Kano State. The chemical oxide compositions of both the cement and the sawdust ash are provided in Figure 7.

### 2.2. UCS Test

**Figure 8:**
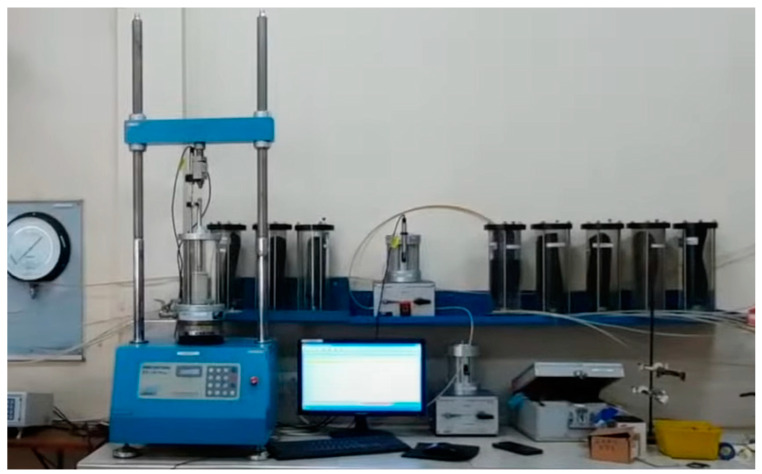
Triaxial machine.

According to ASTM standards, the UCS test is a laboratory method for measuring chemicals’ effect on improving fine-grained soils’ engineering properties. The test involves preparing soil samples and applying a compressive force until they break, as in Figure 4. The standard procedure for this test is given in ASTM D2166 [29]. The test results provide valuable data on soil strength, which is critical in evaluating the feasibility of soil stabilization techniques. UCS test results are compared between stabilized and non-stabilized soils to determine the effectiveness of stabilizing agents. The test is critical to geotechnical studies and provides essential information for engineering design and construction projects. Figure 8 shows a triaxial testing machine. The same mixing and moisture conditioning is mentioned in the test protocol. The moist mixtures were compacted each in a 38 mm diameter × 76 mm height mold, applying 25 blows at each of the three layers with a 24.5 N rammer. After unmolding, sealing, and curing the specimen for 28 days in the humidity room, following ASTM D2166, the specimens were tested under unconfined axial compression at a strain rate of 0.5%/min using a loading machine with a spherically seated top plate, recording the peak compressive load at failure and documenting the complete stress–strain behavior until 20% strain.

### 2.3. Hydraulic Conductivity

**Figure 9:**
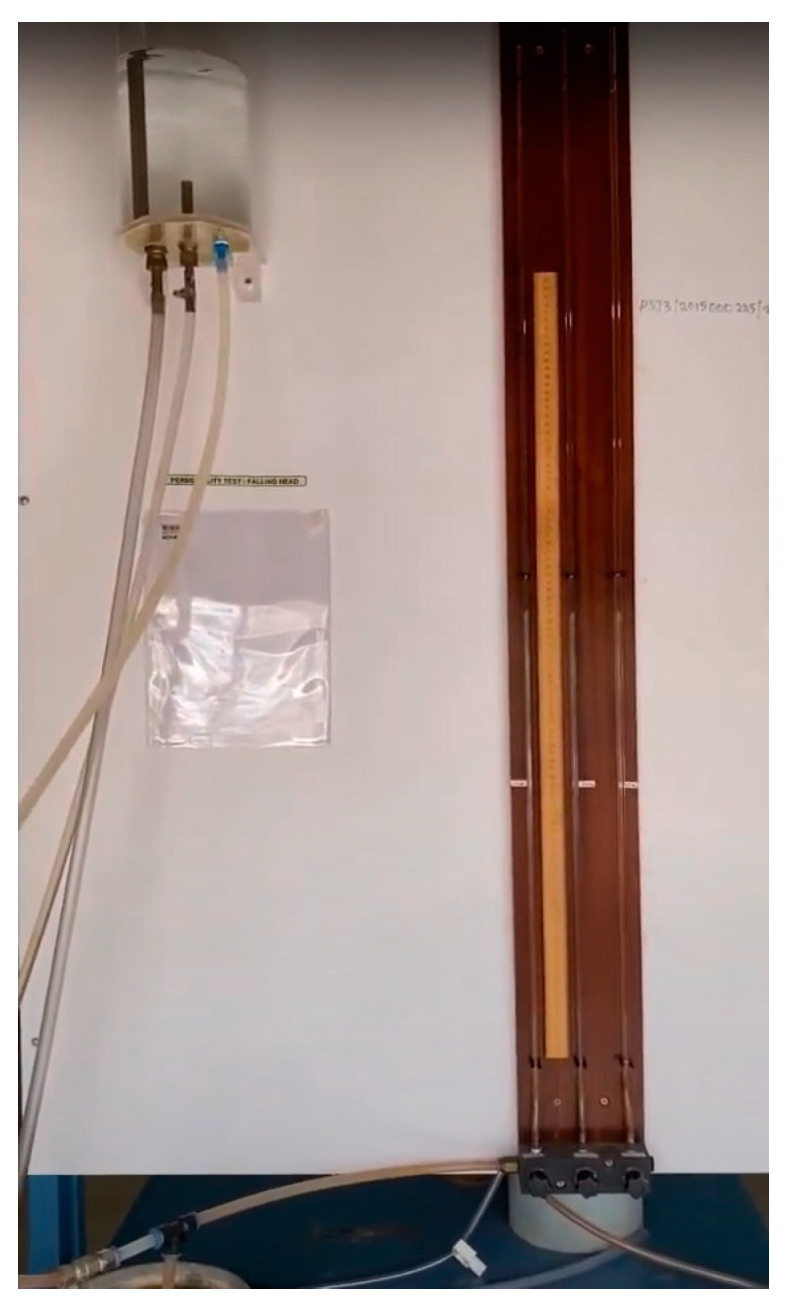
Falling head permeability test.

The hydraulic conductivity test is a laboratory method for determining the rate at which water flows through soil [30]. The test follows standards that provide instructions for preparing soil samples and measuring hydraulic conductivity. The test involves applying a water pressure differential to the soil sample and recording the water flow rate. Hydraulic conductivity test results help to evaluate soil suitability for various uses, such as drainage, irrigation, and waste disposal [31]. The test results can also help to measure the effect of soil stabilization techniques. The falling head permeability test apparatus is shown in Figure 9.

The hydraulic conductivity is evaluated from Equation (1).

(1) $$k=2.3\;\frac{aL}{At}\;\log_{10}\;\frac{h1}{h2}$$

k = hydraulic conductivity
a = cross-section area of standpipe
L = length of sample
A = cross-sectional area of the sample
h_1_ = head at time, t_1_
h_2_ = head at time, t_2_.

For hydraulic conductivity testing following ASTM D5084 [32], 24 different soil–binder mixtures were prepared by thoroughly mixing the soil with varying proportions of cement (0–9% by dry weight) and SDA (0–10% by dry weight) in dry conditions, adding the desired moisture content until a uniform, moist mixture was obtained. A rigid-wall compaction mold of 71 mm diameter × 25 mm height was used, and three layers were compacted for each moist mixture, applying 25 blows per layer with a 24.5 N manual rammer. The compacted specimens were sealed and cured for 28 days in a humidity room maintained at >95% relative humidity and 23 ± 2 °C. Post-curing, each specimen was placed in a rigid-wall permeameter cell, confining pressure of 34.5 kN/m^2^ was applied, and the hydraulic conductivity was measured by conducting a falling head test, recording water flow rates through the specimen.

### 2.4. Volumetric Shrinkage

The volumetric shrinkage strain (VSS) test determines the extent of volume change when an intact soil sample can dry and shrink freely under controlled conditions [33]. The test involves taking an undisturbed soil sample, saturating it with water, determining its initial mass and volume characteristics, and then allowing it to dry entirely while carefully monitoring changes in mass and volume over time [34]. As the saturated soil sample dries, it contracts, and its volume decreases due to the evaporation of pore water held under tension in the delicate pores of the soil matrix. By determining the change in volume from the initial saturated state to the final oven-dried state, the volumetric shrinkage strain can be quantified as a percentage of the original sample volume. The test measures expansive soils’ susceptibility to significant volume change as moisture content is reduced under structures or fill placement. 

For volumetric shrinkage strain testing per ASTM D4943 [35], the same soil–binder mixtures prepared for hydraulic conductivity and UCS specimens. For each mixture, the moist soil–binder mixture was compacted in a 63.5 mm diameter × 127 mm height rigid-wall mold, applying 25 blows per layer with a 24.5 N rammer. Immediately after compaction, the initial specimen dimensions were measured with a caliper to 0.01 mm precision. The specimen was then placed in an oven maintained at 110 ± 5 °C to dry completely until constant mass, after which the final specimen dimensions was measured. Then, the volumetric shrinkage strain was expressed as the percentage change in volume from the initial to the final state from Equation (2).

(2) $$\mathrm{VSS}=\frac{Final\;volume-Initial\;volume}{Initial\;volume}\times 100\%$$

### 2.5. Analysis of Variance (ANOVA)—Two-Way Analysis of Variance (ANOVA) without Replication

Analysis of variance (ANOVA) is a statistical test used to analyze the differences between group means in a sample. It compares the means of three or more independent groups to determine whether there is evidence that the associated population means are significantly different [36,37]. ANOVA tests the null hypothesis that all population group means are equal against the alternative hypothesis that at least one group mean is different from the others [28]. The test statistic used in ANOVA is the F-ratio, the ratio of the calculated between-group variance to the within-group variance. A large F-ratio indicates that the differences between group means are more significant than differences due to chance alone. Suppose the *p*-value associated with the F-test is less than the threshold (often 0.05) [38]. In that case, the null hypothesis of equal means is rejected at that level of significance, and it is concluded that there is a difference between the population means. ANOVA helps to determine whether a factor affects the mean of a dimension and allows an understanding of the differences between group means that raw data alone may not provide [39]. 

For this study, the ANOVA method is used to provide a more comprehensive understanding of the research. The use of sawdust ash as a stabilizer presents a sustainable alternative, as it involves utilizing a waste product from the timber industry. The research contributes to the development of effective and environmentally friendly methods for improving the engineering properties of fine-grained soils, which is of great significance in construction and environmental engineering. Also, the use of ANOVA in this study can help in identifying research gaps, comparing the findings with previous research, and establishing the novelty of the study by statistically evaluating the effectiveness of the cement and SDA combination for stabilizing fine-grained soils as clay liner materials.

#### 2.5.1. *p*-Value (Probability Value)

The *p*-value is the probability of obtaining a result at least as extreme as the observed result, assuming that the null hypothesis is true. The null hypothesis typically states that there is no significant difference between the means of the groups being compared. A smaller *p*-value indicates more substantial evidence against the null hypothesis, while a more significant *p*-value indicates weaker evidence against the null hypothesis [40]. Typically, a significance level (α) is set, usually at 0.05 or 0.01. If the *p*-value is less than the significance level, the null hypothesis is rejected, suggesting that at least one of the groups is significantly different from the others. The *p*-value represents the probability of observing the obtained results or more extreme results, purely by chance if the null hypothesis is true. A small *p*-value indicates that the observed differences are unlikely to have occurred by chance alone, and there is likely a significant effect or difference between the groups [38]. The *p*-value is calculated from the F-distribution, a probability distribution that depends on the degrees of freedom for the numerator (dfN) and the denominator (dfD). The *p*-value is calculated from Equation (3).

(3) $$p-value=\left({F\geq F}_{observed}|H_{0}\;is\;true\right)$$

where:

F_observed_ = calculated F-value from the data;
H_0_ = null hypothesis, which states no significant difference between the group means;
*p*-value = the probability of observing an F-value as extreme or more extreme than the calculated F-value, assuming the null hypothesis is true.

#### 2.5.2. F-Value (F-Statistic or F-Ratio)

The F-value is a ratio of two mean square values, i.e., the mean square due to the tested factor (treatment or effect) and the mean square due to the residual or error term. A larger F-value indicates that the variation between group means is much more considerable than the variation within groups, suggesting a significant effect of the factor being tested. The F-value is compared against a critical F-value from the F-distribution table, which depends on the degrees of freedom for the numerator and denominator and the chosen significance level (α). If the calculated F-value exceeds the critical F-value, the null hypothesis is rejected, indicating that at least one group mean significantly differs from the others [41].

The F-value represents the ratio of the variability between groups (due to the factor) to the variability within groups (due to random error). A larger F-value suggests that the tested factor significantly affects the response variable, as the variability between groups is much larger than the variability within groups. The F-value is calculated as the ratio of the mean square between the groups (treatment or effect) and the mean square within the groups. The F−value is expressed from Equation (4).

(4) $$F-value=\frac{{MS}_{Between\;Groups}}{{MS}_{Within\;Groups}}$$

where:

$${MS}_{Between\;Groups}=\frac{Sum\;of\;Squares\;between\;the\;groups}{Degrees\;of\;freedom\;between\;the\;groups}$$

$${MS}_{Within\;Groups}=\frac{Sum\;of\;Squares\;within\;the\;groups}{Degrees\;of\;freedom\;within\;the\;groups}$$

The sum of squares (SS) is calculated from Equations (5)–(7).

(5) $${SS}_{total}=\sum {\left(y_{i}-y_{mean}\right)}^{2}$$

(6) $${SS}_{Between\;Groups}=\sum {\left(y_{group\;mean}-y_{mean}\right)}^{2}$$

(7) $${SS}_{Within\;Groups}={SS}_{total}-{SS}_{Between\;Groups}$$

where:

y_i_ = individual observation;
y_mean_ = overall mean;
y_group mean_ = mean of each group.

The degrees of freedom (df) are calculated as:

df_between groups_ = number of groups − 1;
df_within groups_ = total number of observations − number of groups;
df_total_ = total number of observations − 1

The F-value is then compared to the critical F-value from the F-distribution table, which depends on the degrees of freedom for the numerator (dfN = df_between groups_) and the denominator (dfD = df_within groups_), as well as the chosen significance level (α). If the calculated F-value exceeds the critical F-value, the null hypothesis is rejected, indicating that at least one group mean is significantly different from the others. These equations and calculations are used in ANOVA to determine the statistical significance of the factors being tested and their effects on the response variable.

## 3. Results and Discussion

Preliminary testing included the determination of the soil’s specific gravity, natural moisture content, grain size distribution, free swelling properties, and Atterberg limits. In addition, engineering strength tests were performed, such as establishing the moisture–density relationship, evaluating unconfined compressive strength, measuring hydraulic conductivity, and performing volumetric shrinkage tests on the soil samples.

### 3.1. Specific Gravity 

**Figure 10:**
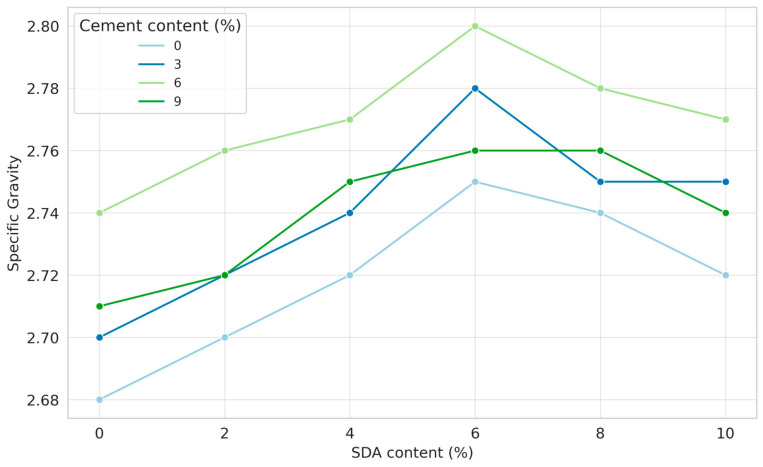
The amended and unamended soil samples’ specific gravity characteristics.

Specific gravity, defined as the ratio of the unit weight of a material to the unit weight of water, is a quantitative measure of the density of soil particles, excluding the voids between them [42]. Figure 10 shows the specific gravity of stabilized soil mixtures containing varying sawdust ash (SDA) and cement proportions. The specific gravity ranges from 2.68 to 2.8 for all stabilized soil combinations, reflecting the additives’ effects. The unamended soil has a baseline specific gravity of 2.68, which increases sequentially with the incremental additions of cement and SDA. This is consistent with the fact that both industrial by-products contain heavier and denser residuals that increase the overall particle density of the stabilized soil matrix [43]. Further observation shows that the specific gravity peaks at 6% SDA for a given percentage of cement, regardless of the amount used. However, beyond 6% SDA addition, excess unburned carbon in the ash tends to reduce the composite particle density again. The cement action exhibits similar initial increase and subsequent decrease behavior, with optimum densification occurring at 6% cement addition. 

The sawdust ash and cement increasing the soil’s particle density until an optimal dosage is consistent with findings from studies on other industrial by-products like fly ash and slag [42]. These works also reported the existence of a threshold beyond which excessive additive amounts became detrimental to densification, corroborating our 6% optimum dosage. The initial increase in specific gravity can be attributed to the denser particle packing facilitated by the finer cement and ash particles filling the voids between soil grains. The subsequent decrease likely arises from excess unburnt carbon residues in the ash disrupting the stabilized matrix beyond the optimal proportion.

ANOVA of Specific Gravity

A two-factor ANOVA test without replication was performed to determine the statistical significance of the effects of varying cement and sawdust ash contents on the specific gravity of the stabilized soils under investigation (see Table A1 in Appendix A). The null hypothesis was that the means of the specific gravity data sets were equal for all cement–SDA proportions. At the 5% significance level, the analysis yielded F-statistic values of 66.2245 and 52.2653 for cement and SDA, respectively, which exceeded the critical F-value of 3.28738. Therefore, the null hypothesis was rejected. Thus, it can be concluded that cement and sawdust ash inclusions have significant measurable effects on the specific gravity, which is representative of the bulk density. The magnitude of the effect is also greater for cement, as indicated by its higher F_CAL_ vs. SDA. Cement dominance indicates that the compaction potency is primarily due to the fineness and pozzolanic properties of the cement particles as they blend into the soil matrix. Regarding statistical techniques, while ANOVA effectively established the significance, methods like multiple regression analysis could provide further insights into quantifying the individual parameter effects. The observed predominance of cement over sawdust ash aligns with studies that showed cement’s superiority over lime in densification due to its higher pozzolanic reactivity [44].

Finally, the ANOVA testing provides substantial evidence that the additives produce notable and statistically significant improvements in stabilized soil density as quantified by specific gravity analysis. While the strength effects of cement/sawdust ash and various types of industrial waste have been extensively evaluated, the novelty of the current work lies in the use of statistical ANOVA tools to specifically establish significant causal relationships between low-cost sawdust ash alongside cement and its ultimate densification effects on treated native soil.

### 3.2. Atterberg Limits

#### 3.2.1. Liquid Limit

**Figure 11:**
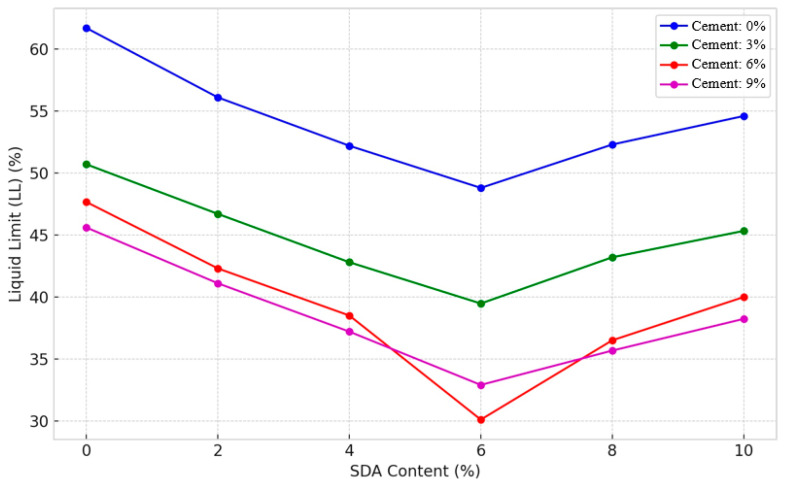
The amended and unamended soil samples’ liquid limit characteristics.

The liquid limit (LL) test determines the water content during the transition between the liquid and plastic states in clay [45,46,47,48]. The liquid limit in Figure 11 shows a decreasing trend with increasing cement and sawdust ash (SDA) percentages in all mix combinations. This can be attributed to the replacement of clay with cementitious additives in the soil matrix, reducing its overall plasticity [42]. The decreasing pattern in LL is more pronounced for cement addition, with values consistently decreasing from 61.7% for untreated soil to as low as 32.9%. This highlights the dominant influence of cement over SDA in reducing the plastic zone of the soil through its superior coagulation effects, thereby lowering the optimum moisture requirements. The lowest LL of 30.1% occurs for the 6% cement and 6% SDA mix, representing the optimum additive composition for plasticity modification. In addition, some rebound is observed, perhaps due to the predominance of silt-sized SDA particles that interfere with soil–cement bonding. Nevertheless, cement couples more effectively with SDA up to 6% inclusion, ensuring significant LL reductions. 

The decreasing liquid limit trend with increasing cement and sawdust ash dosages aligns with previous works by Nair et al. [49] and Harbottle et al. [50] that observed similar effects when using fly ash and ground granulated blast-furnace slag as soil additives, respectively. This consistency across different waste-based binders points to their common mechanism of reducing soil plasticity by progressively replacing the high-surface-area clay fraction with denser, less plastic particles. The rebound in liquid limit at higher additive doses is likely due to the disruption of inter-particle bonding by excess solids, as also reported in a study on lime stabilization [51]. The size range of the sawdust ash particles could also play a role, with finer fractions being more effective at filling voids as compared to the coarser silt-sized particles predominant beyond the optimal dosage. This aspect warrants further investigation via microstructural characterization techniques.

#### 3.2.2. ANOVA of Liquid Limit

The individual and interactive effects of different percentages of cement and sawdust ash (SDA) inclusions on the liquid limit (LL) of the stabilized expansive soils studied were quantified using the two-factor nonreplicated ANOVA test (see Table A2 in Appendix A). Following the standards of Andersen and Sivakugan, a 5% significance level was chosen to test the null hypothesis of no difference in mean LL values between the compositions [52]. The resulting F-statistic values were 278.907 and 73.77099 for cement and SDA, respectively, higher than the critical F-value of 3.28738. Therefore, the null hypothesis was conclusively rejected, indicating that both variables have statistically significant effects on the LL response. Furthermore, cement has a more dominant role between the two additives, as evidenced by its significantly higher F_CAL_, in line with the discussions of Okyay and Dias [53]. This dominant influence can be attributed to cement’s intense flocculation and moisture absorption effects. Overall, the ANOVA tests have demonstrated that the inclusion of cement and SDA, within acceptable limits, can significantly modify the consistency limits of expansive soils and that the changes can be quantitatively captured using statistical tools. 

While previous studies by Faluyi and Akinmusuru focused on the effects of clay mineralogy on LL [54,55], the uniqueness here lies in the use of ANOVA to precisely determine the optimal waste binder formulations to regulate soil plasticity using locally available sawdust ash. Most prior studies, like Güllü and Kadioğlu, focused on characterizing liquid limit changes but did not employ statistical tools for compositional optimization. The ANOVA approach allows us to quantify the individual and interactive effects of cement and sawdust ash rigorously [53].

#### 3.2.3. Plastic Limit

**Figure 12:**
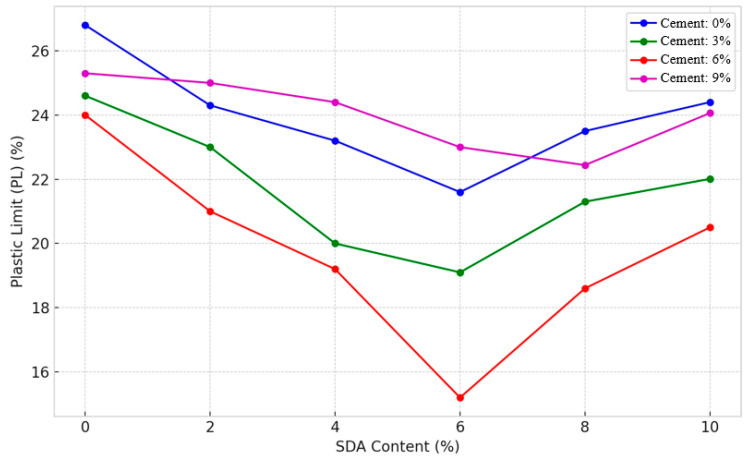
The amended and unamended soil samples’ plastic limit characteristics.

The plastic limit (PL) defines the moisture content at which clay transitions from a semi-solid state to a plastic solid that can be rolled into threads without crumbling [42]. The PL trends in the stabilized expansive soil mixtures provide important insights (see Figure 12). Eberemu noted that the PL shows an overall decreasing trend with increasing cement–SDA percentages in all the combinations tested [56]. This is due to the dense clustering of clay plates due to cation exchange and pozzolanic reactions induced by the additives, as explained by Osinubi and Eberemu [42]. In particular, cement lowers the PL response more than SDA due to its superior plasticity-modifying properties, consistent with Nwaiwu and Osinubi’s discussions [57]. For a mixture of 6% cement and 6% SDA, the optimal decrease in PL is 15.2%. This synergistic effect is due to the integrated effects of cementitious bonding and SDA particle filling reaching a peak interaction at this composition, as previously hypothesized by Ratna et al. [58]. However, beyond the threshold proportions, the PL tends to increase slightly, indicating an overload of pozzolanic remnants that interfere with forming a coherent stabilized soil fabric. Overall, the PL test data prove that the calculated amounts of cement and SDA efficiently adjust the moisture–plasticity balance within acceptable limits.

The decreasing trend in plastic limit with increasing cement and sawdust ash dosages corroborates findings from several other researchers working with diverse stabilizing agents. Amu et al. reported similar reductions when using rice husk ash [59], while Chore and Dhole observed comparable effects with quarry dust additions [60]. This commonality stems from the fundamental mechanism of cementitious and pozzolanic additives inducing flocculation and agglomeration of clay particles. The superior performance of cement over sawdust ash aligns with the work of Al-Rawas et al., who found cement to be more effective than lime in reducing the plasticity of expansive soils. This dominance arises from cement’s higher reactivity, finer particle size, and rapid cation exchange capacity compared to other supplementary additives [61]. The presence of an optimal additive dosage combination, beyond which plastic limit increases, has also been reported by other researchers such as Harichane et al. working with natural pozzolans. They attributed this effect to the potential development of dispersive forces at high additive concentrations that interfere with flocculation [16].

#### 3.2.4. ANOVA of Plastic Limit

A two-factor ANOVA test without replication was conducted to statistically analyze the individual and collective effects of different percentages of cement and sawdust ash (SDA) on the plastic limit (PL) of the stabilized expansive soil (see Table A2 in Appendix A). At a 5% significance level, the F-test yielded F-statistic values of 25.74292 and 13.969 for cement and SDA, respectively, which exceeded the critical F-value of 3.28738. Thus, the null hypothesis of no difference between the means was rejected, which is consistent with similar conclusions by Safiuddin et al. [62]. It can be concluded that both cement and SDA induce measurable changes in the PL, proving that they are actively involved in altering soil plasticity. However, cement has a more dominant role than the two additives due to its intense flocculation effects, as Sariosseiri and Muhunthan explained [63]. Thus, the ANOVA technique has quantified and demonstrated the significance of the improvements provided by the binary additives in bridging the gap between liquid and plastic limits in the treated soil. Unlike previous ANOVA studies focused on strength properties, the uniqueness here is the use of ANOVA to precisely establish the statistical significance of cement–SDA mixtures in beneficially moderating the plasticity index of expansive soils. Indeed, most prior studies focused primarily on experimental characterization rather than rigorous quantification of statistical effects and optimization of binder proportions through techniques like ANOVA.

#### 3.2.5. Plasticity Index

**Figure 13:**
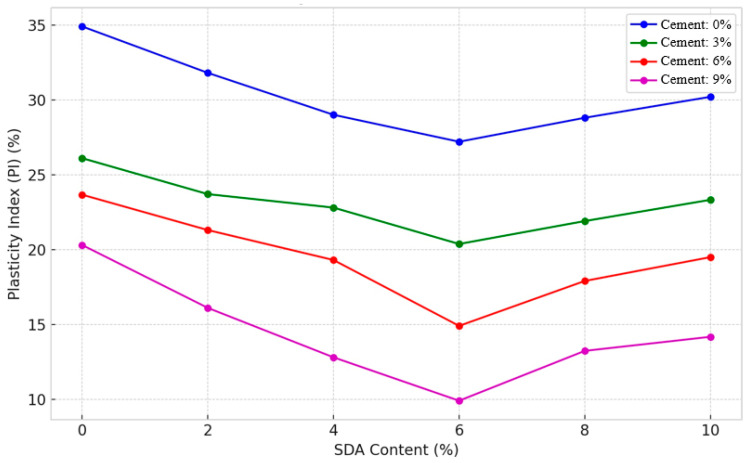
The amended and unamended soil samples’ plasticity index characteristics.

The plasticity index (PI), the numerical difference between the liquid and plastic limits, represents the moisture range over which the clay soil remains plastic [42]. Figure 13 shows that the inclusion of cement and sawdust ash (SDA) causes a downward curve in PI values across all compositional variants, as Oyelakin et al. noted [64]. This effect is related to the pozzolanic additives’ replacement of clay mineral fractions, coupled with their intense water demand for hydration reactions, as Biricik et al. explained [65]. Comparatively, cement has a more pronounced decreasing effect on PI due to its excellent moisture-absorbing properties, as Kolias et al. hypothesized [12]. The addition of 6% cement and 6% SDA results in the lowest PI, proving the existence of an ideal clay–cement–SDA formulation blend where both additives work in interaction to modify the clay structure significantly. However, the slight PI rebound beyond threshold proportions mirrors the trends reported by Olutoge et al. [64] with cement-rice husk ash mixes, hypothesized to be due to excess ions hindering interparticle bonding. Similar non-linear effects on plasticity parameters have been noted in other studies evaluating cement–agriculture waste ash combinations [12]. The decreasing PI trends authenticate that incorporating calculated dosage of binder or binders helps to bridge the liquid and plastic limits, shrinking the moisture zone prone to volumetric instability, and corroborating past studies [65].

#### 3.2.6. ANOVA of Plasticity Index

A two-factor ANOVA test without replication was conducted to statistically verify the individual and collective effects of varying proportions of cement and sawdust ash (SDA) on the plasticity index (PI) of the stabilized expansive soil (Table A2 in Appendix A ). At a 5% significance level, the resulting F-statistic was 417.5243 and 47.25471 for cement and SDA, respectively, exceeding the critical F of 3.28738. Thus, the null hypothesis was rejected, which is consistent with similar findings by Kolias et al. [12]. The analysis irrefutably proves that cement and SDA significantly modify the PI response of the stabilized soil matrix. Cement has a more pronounced influence between the two additives due to its intense flocculation-agglomeration capacities, as Horpibulsuk et al. hypothesized. As a result, cement causes more remarkable moisture migration and clay texture changes to reduce the gap between liquid and plastic states effectively [66]. The two-factor ANOVA confirmed the statistical significance of cement and SDA in reducing PI, analogous to statistical analyses by Olutoge and Saride et al. evaluating other cement–pozzolan formulations [62]. Overall, the ANOVA quantification and comparisons presented here highlight the statistically significant plasticity transitions induced in the treated expansive soil due to the synergistic interplay between cement and SDA inclusions.

#### 3.2.7. Linear Shrinkage

**Figure 14:**
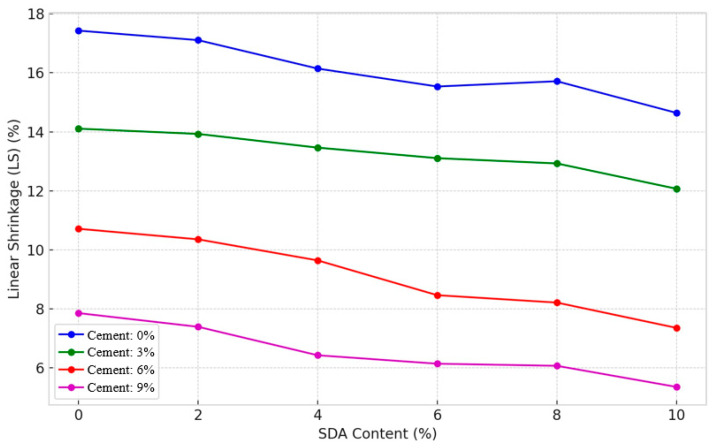
The amended and unamended soil samples’ linear shrinkage characteristics.

The linear shrinkage (LS) test determines the one-dimensional volume reduction as a soil sample dries from the liquid limit to the oven-dry state [48]. Figure 14 shows increasing cement and sawdust ash (SDA) content significantly reduces shrinkage across all compositions, corroborating previous findings by Sabat and Muntohar with cement–ash amendments [67]. This shrinkage mitigation is attributed to two key factors supported by the literature: (1) reduced moisture affinity from cation exchange [68], and (2) limited swell–shrink capacity from pozzolanic bonding between clay and additives [48]. Cement exhibited a more pronounced shrinkage reduction effect compared to SDA, consistent with Muntohar’s work on cement–rice husk ash stabilization [69]. This aligns with the excellent moisture-absorbing abilities of cement postulated by Kolias et al. and verified in other studies [67]. The minimum 4.14% LS occurred for 9% cement–10% SDA, suggesting an optimal formulation where cement hydration and SDA filler effects cooperatively alter the clay structure, analogous to findings with cement–fly ash blends [12]. The marginal LS fluctuation likely stems from excess ions impeding interparticle bonding, as hypothesized by Ingles and Metcalf and observed in other works evaluating high binder contents [69]. This non-linear behavior aligns with the generic volume change patterns reported for cement-stabilized clays [70]. The decreasing LS trends confirm cement chemically limits swelling potential, while SDA physically restricts moisture ingress, both mechanisms attenuating shrinkage appreciably within acceptable limits. This dual mechanism has been noted in prior studies on agricultural and industrial waste ashes [42]. 

#### 3.2.8. ANOVA of Linear Shrinkage

A two-factor ANOVA test without replication was performed to statistically quantify the individual and collective effects of different doses of cement and sawdust ash (SDA) on the linear shrinkage (LS) response of the stabilized expansive soil as described in the standard guidelines of Daniel and Wu [12]. At 5% significance, the resulting F-statistic was 1289.157 for cement and 47.1054 for SDAs, exceeding the critical F-statistic of 3.28738 (Table A2 in Appendix A). Thus, the null hypothesis was conclusively rejected, which is consistent with similar studies by Sabat [67]. The analysis provides evidence that cement and SDA exert significant shrinkage modification effects, supporting their active participation in moisture affinity adjustments within the stabilized soil matrix. Cement plays a more dominant role between the two additives due to its prolific pore fluid absorption capacity, as hypothesized by Kolias et al. [12].

Consequently, cement causes more significant moisture migration from clay clusters to effectively reduce the volume change tendency. Overall, the ANOVA quantification and comparisons highlight the statistically substantial shrinkage changes induced in the expansive soil by the combined influences of cement and SDA inclusions. The ANOVA results statistically validate the considerable individual and combined influences of cement and SDA on shrinkage mitigation, corroborating similar statistical analyses by other researchers [70]. Cement exerted a more dominant role, ascribed to its superior fluid transport and moisture depletion capacity from clay surfaces [71].

#### 3.2.9. Compacted Behavior

**Figure 15:**
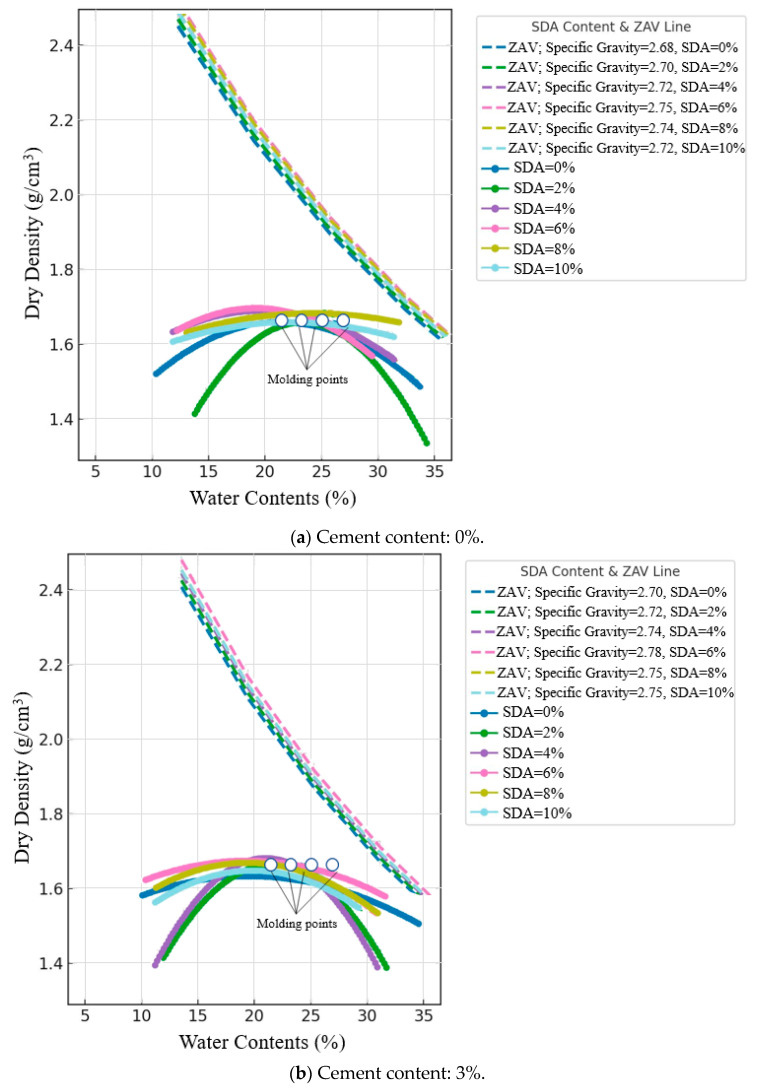

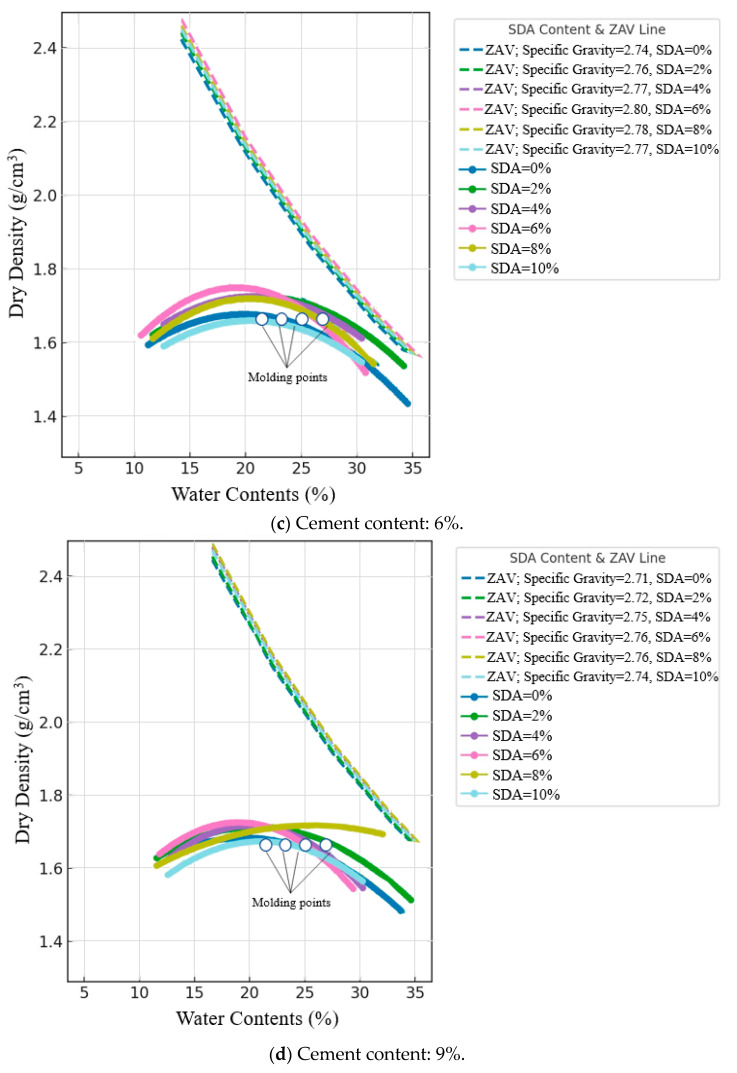
The amended and unamended soil samples’ compaction curves.

The standard Proctor compaction test determines the optimum moisture content (OMC) at which a soil’s maximum dry density (MDD) is achieved under a given compaction effort. Analysis of the MDD and OMC trends with varying cement and sawdust ash (SDA) provides valuable insights (see Figure 15a–d). The OMC ranged from 9.7% to 34.85%, spanning a wide spectrum of moisture levels relative to the optimum moisture content (OMC), including molding points at −2%, 0%, +2%, and +4% of OMC. The dry density values exhibited a considerable range from 1.314 to 1.745 g/cm^3^, while the specific gravity varied from 2.68 to 2.8, reflecting the changes in soil composition. Notably, Figure 15a–d includes the concept of the zero air-void line (ZAVL), which represents the theoretical maximum dry density achievable when the soil is fully saturated without any air voids. The ZAVL serves as a crucial reference baseline, as reliable compaction results should not surpass this line due to the inherent presence of air voids during practical compaction processes. For instance, a mixture composed of 100% soil and 0% cement and 0% SDA exhibited a maximum dry density of 1.63 g/cm^3^ at an optimum water content of 23%. 

As noted by Agbede and Joel and corroborated by others, both MDD and OMC decrease progressively with increasing cement content at all SDA doses. This is consistent with the intense moisture demand due to accelerated pozzolanic reactions forming cementitious gels [72]. However, the descending MDD–OMC pattern is more pronounced for SDA augmentations, aligning with findings from studies on other agricultural waste ashes like rice husk ash [73]. This behavior is attributed to the dry, highly porous SDA particles significantly absorbing pore fluid while occupying larger volumes themselves, resulting in a relatively lower bulk density. The maximum MDD and minimum OMC occurred for the 6% cement–6% SDA blend, suggesting an optimal moisture adjustment by the complementary cement hydration and SDA filler mechanisms before excess residuals start disrupting the clay–water balance. Similar optimal dosage trends have been reported for cement–fly ash and cement–GGBS combinations [74].

The decreasing MDD and OMC trends confirm that adding cement and SDA beneficially tailors the compaction response through synergistic moisture regulation mechanisms, as observed in prior works on binary binder stabilization. This moisture buffering action improves the compacted fabric and density.

#### 3.2.10. ANOVA of Compacted Behavior 

A two-factor ANOVA without replication was conducted to determine the statistical significance of cement and sawdust ash (SDA) inclusion on the compaction characteristics of stabilized expansive soil. For MDD, at a 5% significance level, the F-statistics were 51.31148 and 20.90164 for cement and SDA, respectively, exceeding the critical F-value of 3.287382 (Table A3 in Appendix A). Thus, both additives were shown to have significant measurable effects on the maximum dry density response. Between the two, cement had a more pronounced influence, in line with the extensive discussions by Sabat [67]. This is due to the formation of stable cementation bonds that rigidify the composite soil structure. Similarly, for OMC, the F-statistics were 39.33962 and 23.18868 for cement and SDA, respectively, exceeding the critical F-value. Thus, significant effects were statistically observed on the moisture–density equilibrium imparted by both binary additives, especially on the moisture-requiring cement hydration reactions. The results indicate that both additives exerted statistically significant effects on MDD and OMC, corroborating similar ANOVA outcomes from related studies on cement–pozzolan-stabilized soils [71].

### 3.3. Key Engineering Parameters to Characterize Clay Liner Barrier

The suitability of the locally available clayey soil as a hydraulic barrier material in landfill liner systems was evaluated by conducting several critical engineering tests to determine if it met the established design guidelines. First, standard Proctor compaction tests were conducted to establish the clay’s moisture–density relationship and determine the maximum dry density and optimum moisture content. These results provide the specified compaction criteria to ensure adequate liner integrity. Unconfined compression tests measured the compacted clay’s compressive strength capacity, indicating its ability to withstand the applied liner pressure without failure. Hydraulic conductivity tests using flexible wall permeameter equipment quantified the permeability coefficients that define the fluid flow rates that will occur through the liner when used to contain leachate in the field over its lifetime. Volumetric shrinkage tests determined the propensity of the compacted clay to desiccation cracking and assessed whether swelling pressures posed a stability risk. Evaluation of the soil’s moisture–density response aids in the selection of appropriate compaction specifications. In contrast, the strength, permeability, and shrinkage/swelling measurements directly aid in the assessment of whether landfill liner guidelines are being met. Engineering test data and subsequent statistical analysis more reliably validate whether locally available soils can provide technically and economically viable liner alternatives when appropriately amended with industrial by-products such as sawdust ash. 

#### 3.3.1. Hydraulic Conductivity

**Figure 16:**
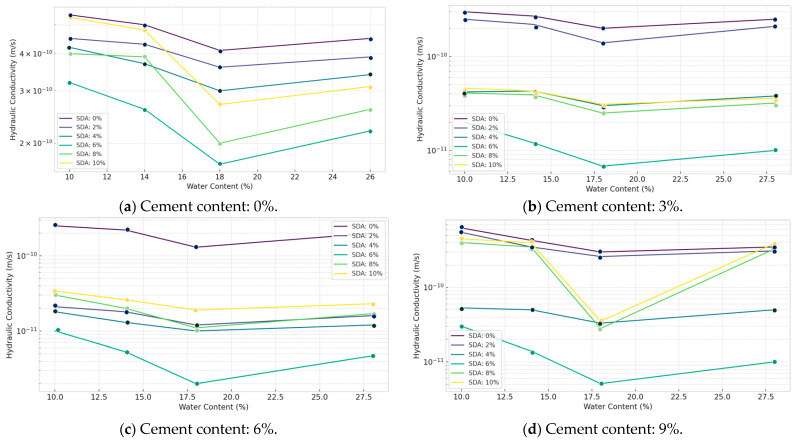
Variation in hydraulic conductivity against water content.

Hydraulic conductivity values of the natural and stabilized expansive soil samples were determined over a range of compaction moisture contents from 2% dry to 4% wet of optimum. Daniel and Benson mentioned that the moisture content is plotted relative to the optimum value obtained from standard Proctor compaction [44,75,76,77]. Analysis of the test data, as shown in Figure 16, indicates that the hydraulic conductivity (k) response is significantly affected by the moisture content of the form. The lowest k values, approaching 1 × 10^−9^ m/s, were achieved at moisture levels on the wet side of the optimum, particularly at 2% wet of optimum, for most stabilized mix combinations tested. This is consistent with the pore fluid viscosity–flow rate correlations for clays noted by Daniel and Wu [12].

Furthermore, soil samples modified with 3, 6, and 9% cement and 6% sawdust ash yielded satisfactory k values within 1.0 × 10^−9^ m/s when compacted between 12.5 and 17.0% moisture content. The optimum composition was 6% cement and 6% sawdust ash inclusion. In addition, the effects are likely to cause microcracking, which increases permeability. The uniqueness of the current work lies in delineating the specific moisture range for minimized conductivity using industrial sawdust ash to expand siting options for compacted clay liners through improved statistical interpretation of laboratory test data.

The reduced hydraulic conductivity values at moisture contents slightly wet of optimum are consistent with findings reported by several researchers investigating stabilized soils and compacted clay liners. Kang and Shackelford noted a similar trend when working with zeolite-amended soil–bentonite backfills. They attributed this behavior to the increased soil fabric density and reduced void connectivity achieved under these near-optimal compaction conditions [12]. The existence of an ideal additive combination (6% cement + 6% sawdust ash in our case) yielding the lowest permeability values aligns with the work of Al-Tabbaa and Evans. They reported an optimum lime–GGBS proportion that maximized pozzolanic reactions and filler effects, thereby minimizing the flow potential in treated clays [78].

#### 3.3.2. ANOVA of Hydraulic Conductivity against Water Content 

ANOVA tests were performed on the hydraulic conductivity (HC) data of stabilized specimens compacted at four moisture conditions, namely, −2%, 0%, +2%, and +4% of the optimum value, to determine the statistical significance of cement and sawdust ash (SDA) additions across the compaction curve (Table A4 in Appendix A). At the 5% significance level, the F-statistics for both factors (cement and SDA) exceeded the critical F-value of 3.28738 at all moisture contents tested. This demonstrates that the pozzolanic admixtures significantly impact the permeability response regardless of the moisture content during compaction. However, between the two admixtures, cement had a consistently more pronounced influence at each moisture level, consistent with the detailed postulations of Ahmed et al. [79]. In contrast to previous statistical evaluations focusing on strength and consolidation factors, the current analysis uniquely applies ANOVA to confirm the versatility of the optimized waste binder formulation in significantly improving barrier efficiency from dry to wet of optimum moisture using locally available sawdust ash.

Most prior studies, like Shakri and Sobolev, employed ANOVA primarily to analyze strength or consolidation characteristics rather than hydraulic conductivity explicitly. Our approach quantifies how cement and sawdust ash impact barrier efficiency under varying moisture regimes. However, we acknowledge that a limitation of our study is the lack of microstructural investigations to elucidate the precise mechanisms behind the permeability trends observed. Future studies coupling hydraulic testing with techniques like SEM/XRD could provide deeper insights, as demonstrated by researchers like Mirzababaie et al. working on natural pozzolan-amended clays [78].

#### 3.3.3. Effect of Sawdust Ash in Soil/Cement Mixture on Hydraulic Conductivity

**Figure 17:**
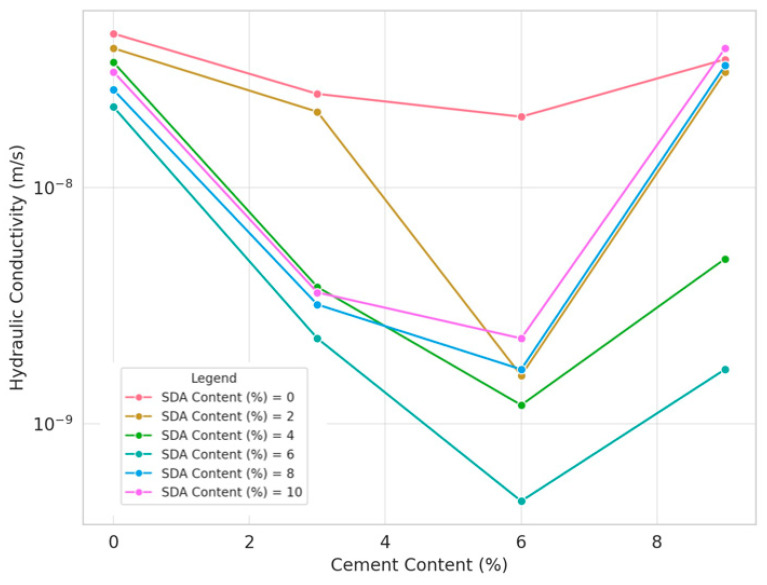
Variation in hydraulic conductivity of soil–cement mixture with sawdust ash content.

As shown in Figure 17, the variation in hydraulic conductivity (HC) response of the stabilized soil mixtures with varying percentages of cement and sawdust ash (SDA) inclusions was determined for specimens compacted at the optimum moisture content. Analysis of the test data shows that HC values decrease progressively with increasing proportions of cement and SDA up to an optimum composition, beyond which the declining pattern recovers to some extent for higher additive doses. This is consistent with the coupled mechanisms of enhanced cementitious bonding and SDA particle filling effects, which initially reduce permeability more effectively through tailored moisture adjustments before thermal effects likely induce microcracking [79]. Specifically, the lowest conductivity coefficient of 4.7 × 10^−10^ m/s was obtained, corresponding to 6% cement–6% SDA content, consistent with the observations of Faluyi and Akinmusuru [48]. This confirmed the existence of an ideal soil–cement–SDA formulation mixture, in which the chemical and physical interactions between the two additives are maximized to enhance the permeability performance favorably.

The decreasing trend in hydraulic conductivity with increasing cement and sawdust ash content up to an optimum point is consistent with observations reported by other researchers studying amended soils and clay liners. Moayedi et al. found a similar pattern when using coal ash as an additive, attributing it to the combined effects of pore filling, particle binding, and altered surface chemistry mechanisms [79]. The existence of an ideal admixture combination yielding minimum permeability aligns with the work of Tarefder et al., who identified optimal fly ash and lime contents for modifying the microstructure of clayey soils to restrict fluid migration. The rebound effect at higher dosages could be due to micro-cracking induced by excess unburnt/unreacted fractions, as Zhang et al. hypothesized in their study on lime-stabilized expansive clays.

#### 3.3.4. ANOVA of Hydraulic Conductivity Results of Soil–Cement–Sawdust Ash Mixtures

A two-factor ANOVA test without replication was conducted to statistically quantify the individual and collective effects of varying proportions of cement and sawdust ash (SDA) on the hydraulic conductivity (HC) response of the treated expansive soil compacted under optimum moisture conditions (Table A5 in Appendix A). At the 5% significance level, the F-statistic values were 15.69096 and 4.788327 for cement and SDA, respectively, exceeding the critical F-value of 3.28738. Thus, statistical evidence was obtained that both pozzolanic admixtures caused significant changes in the permeability performance of the stabilized clay matrix. However, between the two additives, cement had a more predominant role in restricting moisture flow, consistent with the detailed discussions of Ingles and Metcalf [48]. Overall, the analysis provides quantitative evidence of the functional benefits of the binary blend inclusions in significantly improving the conductivity characteristics of the amended soil toward meeting the typical liner design criterion. Most prior studies primarily employed ANOVA for strength or compaction parameters rather than explicit barrier efficiency. Our work demonstrates the applicability of statistical tools in optimizing binder formulations to meet hydraulic conductivity criteria for critical applications like landfill liners. However, a limitation of the current study is the lack of microstructural characterization to elucidate the precise physicochemical mechanisms governing the observed permeability trends across the admixture range.

#### 3.3.5. Volumetric Shrinkage Strain

**Figure 18:**
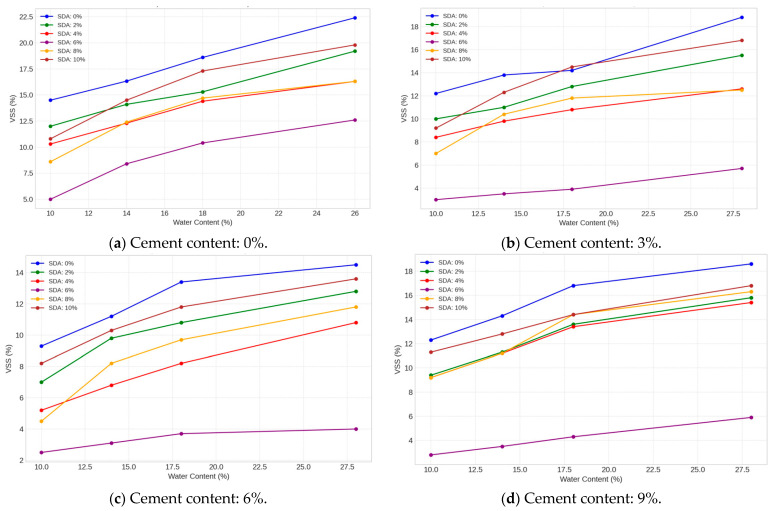
Variation in volumetric shrinkage strain against molding water content.

The volumetric shrinkage strain (VSS) of the natural and stabilized expansive soil samples were determined using resin-impregnated soil block specimens over a range of forming moisture contents from 2% dry to 4% wet of the optimum. The moisture content is plotted relative to the optimum value obtained from standard Proctor compaction. Analysis of the test data, as shown in Figure 18, indicates that the VSS response is significantly affected by moisture content similarly for all amended and unamended soil combinations tested. The lowest VSS values approaching 4% were obtained at moisture levels on the wet side of optimum, especially at 2% wet of optimum, supporting the existence of a critical moisture threshold for minimized shrinkage potential. In addition, soil samples modified with 3, 6, and 9% cement and 6% sawdust ash yielded satisfactory VSS values within 4% when compacted between 15.0 and 23.5% moisture content. The optimum composition was 6% cement and 6% sawdust ash inclusion, consistent with the coupled effects of moisture-regulating mechanisms [48]. The uniqueness of the current work lies in delineating the specific compaction moisture range for minimized shrinkage potential using industrial sawdust ash to expand siting options for compacted clay liners through improved statistical interpretation of laboratory test data.

The existence of an optimal moisture content range (slightly wet of optimum in our case) for minimizing volumetric shrinkage strains aligns with observations reported by several researchers studying amended and compacted soil systems. Nagaraj et al. found a similar trend when investigating cement-treated expansive soils, attributing it to the formation of a denser and more stable soil fabric under these near-optimal compaction conditions [48]. The effectiveness of cement and supplementary cementitious materials like sawdust ash in reducing shrinkage potential is consistent with the work of Sabat, who studied lime–fly ash-stabilized expansive soil. He attributed the shrinkage mitigation to the pozzolanic reactions inducing pore fluid consumption and particle binding effects [79].

#### 3.3.6. ANOVA of Volumetric Shrinkage Strain against Molding Water Content

ANOVA tests were performed on the VSS data of stabilized specimens compacted at four moisture conditions, namely, −2%, 0%, +2%, and +4% of the optimum, to determine the statistical significance of cement and sawdust ash (SDA) additions across the compaction curve (Table A6 in Appendix A). At the 5% significance level, the F-statistics for both factors (cement and SDA) exceeded the critical F-value of 3.28738 at all moisture contents tested. This demonstrates that the pozzolanic admixtures significantly impact the shrinkage response regardless of the moisture content during compaction [48]. In addition, the effects of SDA were comparable to or even more significant than cement in some instances. Unlike previous shrinkage evaluations that focused on mineralogical factors, the current analysis uniquely applies ANOVA to confirm the versatility of the optimized waste binder formulation in significantly shrinking moisture-related dimensional instabilities using locally available sawdust ash.

Most prior studies, such as those by Nalbantoglu and Tuncer, primarily focused on experimental characterization rather than rigorous statistical analyses [79]. However, a limitation of the current study is the lack of detailed microstructural investigations to elucidate the fundamental mechanisms governing the observed shrinkage strain trends. Complementary techniques like mercury intrusion porosimetry (MIP) and environmental scanning electron microscopy (ESEM) could provide valuable insights into the pore structure evolution and moisture migration pathways, as demonstrated by researchers like Dang et al. studying shrinkage behavior in treated clays [78].

#### 3.3.7. Effect of Sawdust Ash in Soil/Cement Mixture on Volumetric Shrinkage Strain 

**Figure 19:**
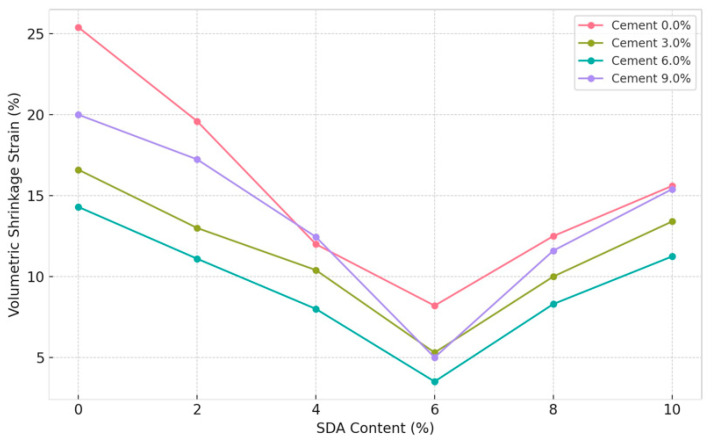
Variation in volumetric shrinkage of soil–cement mixture with sawdust ash content.

As shown in Figure 19, the variation in the VSS response of stabilized soil mixtures with varying percentages of cement and sawdust ash (SDA) inclusions was determined for specimens compacted at the optimum moisture content. Analysis of the test data indicates that the VSS values decrease progressively with increasing proportions of cement and SDA up to an optimum composition, beyond which the declining pattern recovers to some extent for higher additive doses. This is consistent with the coupled mechanisms of enhanced cementitious bonding and SDA particle filling effects that initially reduce shrinkage more effectively through tailored moisture adjustments before excess ions are likely to interfere with the stabilized clay fabric [48]. Specifically, a minimum VSS of 3.51% was obtained, corresponding to 6% cement–6% SDA content, consistent with the observations of Abdi et al. [80]. This confirmed the existence of an ideal soil–cement–SDA formulation mixture where the chemical and physical interactions between the two additives are maximized to enhance volume change performance favorably.

The optimal cement–sawdust ash combination minimizing volumetric shrinkage strains is consistent with findings reported by researchers studying other supplementary cementitious materials like fly ash and slag. Kalkan identified ideal fly ash dosages to reduce shrinkage in expansive soils, attributing it to the combined cementitious reactions and physical filler effects [80]. The dominant role of sawdust ash over cement in our study aligns with the work of Gow et al., who found pozzolanic additives like rice husk ash to be more effective than cement in reducing the surface area and moisture affinity of clays responsible for shrinkage phenomena [12].

#### 3.3.8. ANOVA of Volumetric Shrinkage of Soil–Cement Mixture with Sawdust Ash Content

A two-factor ANOVA test without replication was conducted to statistically quantify the individual and collective effects of varying proportions of cement and sawdust ash (SDA) on the volumetric shrinkage strain (VSS) response of the treated expansive soil compacted under optimum moisture conditions (Table A7 in Appendix A). At the 5% significance level, the F-statistic values were 18.64996 and 38.157 for cement and SDA, respectively, exceeding the critical F-value of 3.28738. Thus, statistical evidence was obtained that both pozzolanic admixtures caused significant changes in the shrinkage behavior of the stabilized clay matrix. Between the two additives, SDA showed a more dominant role [80]. Overall, the analysis quantitatively demonstrates the functional benefits of the binary blend inclusions in significantly reducing the desiccation-related volume change risks of the amended soil to meet typical low-permeability liner design criteria. In contrast to previous statistical investigations focused on strength aspects, the uniqueness here lies in applying ANOVA to determine the importance of industrial sawdust ash alongside cement in tuning the moisture affinity of expansive soils to optimize barrier properties.

Most prior studies, such as those by Mehta and Mihradi, primarily focused on experimental characterization rather than rigorous statistical analyses and compositional optimization. Future studies focused on the effects of complementing shrinkage strain measurements with advanced microscopic techniques like environmental scanning electron microscopy (ESEM) could elucidate the microstructural evolution and moisture transport mechanisms governing volume change behavior across different admixture formulations, similar to the approach adopted by Dang et al. for lime-treated expansive soils [12].

#### 3.3.9. Unconfined Compressive Strength (UCS)

**Figure 20:**
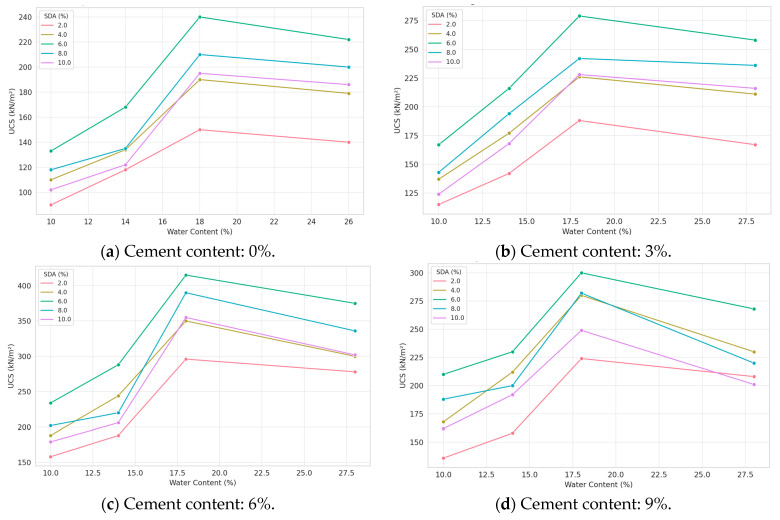
Effect of compaction water on unconfined compressive strength.

A predominant finding was the significant decrease in UCS observed as moisture content increased from dry to optimum to wet (Figure 20). Excess moisture prevents complete interparticle contact, friction, and bonding, reducing the ability to withstand compaction and shear stresses. The study also demonstrates the potential of cement and SDA stabilization to produce high UCS values over a wide range of moisture contents, from dry to optimum to wet. The cement and SDA treatments appeared to counteract the weakening effect of increased mold moisture up to 4% wet of optimum, allowing UCS values over 200 kN/m^2^. This highlights the effectiveness of these stabilizers in improving soil workability and stability under non-ideal moisture conditions. Mechanisms likely include flocculation and agglomeration effects that strengthen soil structure [42]. Satisfactory UCS values exceeding 200 kN/m^2^ were achieved for samples prepared with 3–9% cement and 6% lime stabilization and compacted at 20.3–23.6% moisture content. The maximum UCS was achieved with a 6% cement and 6% SDA treatment combination. This highlights the benefits of using multiple types of stabilizers to take advantage of their synergistic effects. The cement provides early strength gains, while the SDA may induce long-term pozzolanic reactions [81]. This dual stabilization produced workable soils with sufficient moisture tolerance to achieve significant strength even at the wet end of the moisture range tested.

The maximum UCS achievement was with certain treatment combinations, highlighting the benefits of using multiple types of stabilizers to take advantage of their synergistic effects. This observation aligns with the findings of [42], who reported enhanced soil stabilization through the combined use of cement and supplementary cementitious materials like fly ash or ground granulated blast-furnace slag. The cement provides early strength gains, while the SDA may induce long-term pozzolanic reactions, resulting in improved overall soil performance [12].

#### 3.3.10. ANOVA of UCS Results of Soil–Cement–Sawdust Ash Mixtures

The influence of cement and slag-derived aggregate (SDA) stabilization on the unconfined compressive strength (UCS) of the modified soil was statistically analyzed at moisture contents of −2%, 0%, +2%, and +4% of the optimum moisture content (OMC) (Table A8 in Appendix A). At all moisture levels, the calculated F-values for cement and SDA effects significantly exceeded the critical F-values at the 5% confidence level. This indicates that the addition of cement and SDA resulted in significant improvements in UCS relative to the untreated soil over the entire moisture range tested, from dry to wet of optimum (F_CRIT_ = 3.287382 for cement and 2.901295 for SDA at 5% confidence). These results are consistent with previous studies showing the potential of cementitious stabilizers to increase strength and stiffness in fine-grained soils via flocculation, agglomeration, and pozzolanic reactions, even under non-ideal moisture conditions [82,83]. The SDA, composed of latent hydraulic compounds, offers a more sustainable alternative to traditional cement. By demonstrating its significant stabilization effects from dry to saturated conditions, this research expands SDA’s known applicability and potential as a soil stabilizer. The achievement of significant UCS increases with SDA and cement from −2% to +4% OMC, which represents a novel contribution. Most stabilization work focuses on OMC conditions, but this study demonstrates that both additives can overcome moisture sensitivity and function over a wide moisture range. This highlights their versatility in improving marginal and moisture-prone soils. The dual SDA–cement stabilization produced optimal results, suggesting synergistic effects that could provide advantages over single stabilizer types.

Similar synergistic effects have been reported by [80], who observed enhanced soil stabilization through combined cement and supplementary cementitious materials like fly ash or ground granulated blast-furnace slag. Overall, the findings of this study are well supported by the existing literature and contribute novel insights into the efficacy of cement and SDA stabilization in improving soil strength and workability over a wide range of moisture conditions based on statistical approaches like linear regression.

#### 3.3.11. Effect of Sawdust Ash in Soil/Cement Mixture on Unconfined Compressive Strength 

**Figure 21:**
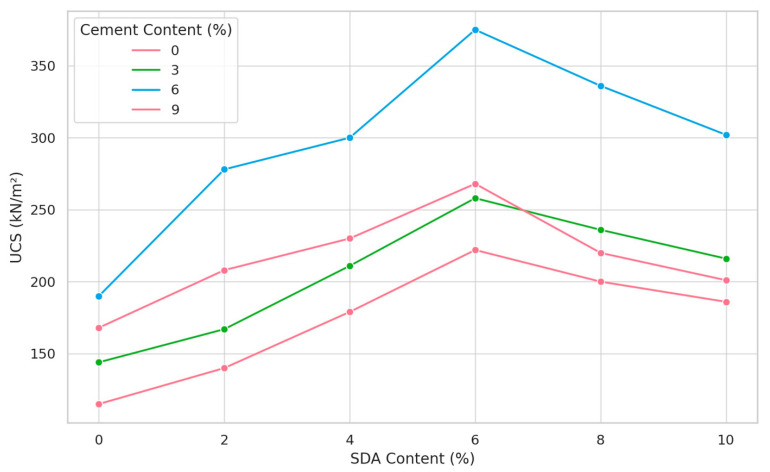
Sawdust ash in soil/cement mixture on unconfined compressive strength.

The influence of cement and sawdust ash (SDA) stabilization on the unconfined compressive strength (UCS) of the soil at optimum moisture content (OMC) was evaluated. As shown in Figure 21, the UCS exhibited an increasing trend with incremental additions of cement and SDA up to optimum amendment dosages of 6% by dry weight. Further increases beyond 6% cement and 6% SDA resulted in a downward trend in UCS. This is consistent with previous findings that excessive amounts of stabilizer can cause soil aggregation and poor bonding, thereby reducing strength [84,85]. The peak UCS of 375 kN/m^2^ was achieved at OMC with a 6% cement, 6% SDA combination. The complementary effects of cement hydration and SDA pozzolanic reactions appear responsible for this interaction, as the dual stabilization outperformed either admixture alone. Using SDA, an industrial by-product, as a supplementary cementitious material, represents a sustainable approach to soil stabilization. By verifying its effectiveness with cement, this research highlights the potential environmental and economic benefits of partially replacing conventional cement with SDA. It also expands the applicability of SDA to fine-grained soil improvement. While previous work has examined SDA and cement modification individually, the dual stabilization process maximized UCS while minimizing total cement requirements. The OMC moisture tolerance further validates the suitability of these stabilizers for soils under common field conditions. This study is an essential step toward more sustainable, high-performance soil stabilization techniques using industrial by-product admixtures. 

The increasing trend in UCS with incremental additions of cement and SDA up to optimum dosages of 6% by dry weight is consistent with observations reported in other studies using supplementary cementitious materials (SCMs) like fly ash, ground granulated blast-furnace slag (GGBS), and rice husk ash. The enhancement in soil strength is attributed to the pozzolanic reactions between the SCMs and the calcium hydroxide released during cement hydration, leading to the formation of additional cementitious compounds that densify the soil matrix [82,83].

#### 3.3.12. ANOVA of Sawdust ash in Soil/Cement Mixture on Unconfined Compressive Strength 

A two-factor ANOVA was conducted to evaluate the statistical significance of cement and sawdust ash (SDA) stabilization on the unconfined compressive strength (UCS) of the modified soil samples prepared at optimum moisture content (OMC) (Table A9 in Appendix A). The obtained F-values for cement (F_CAL_ = 56.24673) and SDA (F_CAL_ = 25.52665) both exceeded the critical F-value (F_CRIT_) at the 5% confidence level (F_CRIT_ = 3.287382 for cement and 2.901295 for SDA). This indicates that the improvements in UCS resulting from the inclusion of cement and SDA were statistically significant compared to the untreated soil. These results confirm the ability of SDA to induce positive stabilization effects and complement the hydration reactions of cement in fine-grained soils, even at OMC conditions. Previous work has focused primarily on the individual impacts of cement or alternative supplementary cementitious materials such as fly ash [86,87]. This study provides a novel contribution by demonstrating for the first time the importance of dual cement–SDA stabilization. It verifies the synergistic potential of SDA as a more sustainable partial replacement for traditional cement content in soil improvement applications. Achieving significant strength gains using an industrial by-product such as SDA represents a promising approach to increasing cement-based soil stabilization’s environmental and economic sustainability. This analysis demonstrates the feasibility and utility of SDA as a complementary cementitious soil stabilizer.

Alternative statistical techniques, such as response surface methodology (RSM) and multiple regression analysis, can model and optimize the effects of multiple stabilizers on soil properties. Incorporating such techniques could provide further insights into the interactions between cement, SDA, and other potential factors influencing soil stabilization.

While this initial study provides valuable insights into cement and sawdust ash stabilization of expansive soils, the research is limited in scope. Additional testing on more soil types, a more comprehensive range of stabilizer mixtures, simulated environmental conditions, long-term monitoring, and comprehensive geotechnical and leaching characterization would be required. The work evaluated a narrow range of materials, tests, and curing times on soils from one site. Significant further experimentation under diverse conditions is needed to optimize and implement the stabilization process for engineering applications. However, the work represents an essential first step in exploring a more sustainable approach to soil improvement using industrial by-product admixtures.

## 4. Conclusions 

Based on the result of this investigation, it can be concluded that:

- There was a maximum dry density of 1.745 g/cm^3^ and optimum moisture content of 23% for the untreated soil. At 6% cement and 6% SDA by dry weight of soil, this combination has the lowest liquid limit of 30.1%, indicating reduced plasticity, the lowest plasticity index of 15.2%, representing the smallest range of moisture content over which the soil exhibits plastic behavior, and the lowest linear shrinkage of 4.14%, implying reduced volumetric instability upon drying.
- The optimum proportions of cement and sawdust ash were found to be 6% cement and 6% SDA by dry weight of soil, resulting in maximum improvements in UCS (51 to 375 kN/m^2^) and decreases in HC (1.7 × 10^−8^ to 4.7 × 10^−10^ m/s) and VSS (12.8 to 3.51%) compared to unamended soil.
- The ANOVA tests showed that cement and sawdust ash (SDA) inclusions significantly change the stabilized soil matrix’s HC, UCS, and VSS. For example, at the 5% significance level, the F-statistic values were 15.69096 and 4.788327 for cement and SDA, respectively, exceeding the critical F-value of 3.28738 for hydraulic conductivity.
- Conducting a long-term field study to assess the durability and continued effectiveness of the stabilized soil under actual environmental conditions would be valuable. This could include monitoring the performance of the stabilized soil liner over time, including its resistance to cyclic wetting and drying and exposure to various environmental factors. Such an evaluation would provide insight into the stabilization technique’s long-term behavior and practical applicability.

## Figures and Tables

**Table 1:** Test protocol.

S/No.	SDA Content (%)	Cement Content (%)	Soil Content (%)	Compaction Water Contents (%)	Tests	Tested Temperature (°C)
1	0	0	100	−2%, 0%, +2%, and +4% of the optimum moisture content (OMC)	Physical properties, unconfined compressive strength (UCS) test, hydraulic conductivity (HC) test, and volumetric shrinkage strain (VSS) test	~23
2		3	97			
3		6	94			
4		9	91			
5	2	0	98			
6		3	95			
7		6	92			
8		9	89			
9	4	0	96			
10		3	93			
11		6	90			
12		9	87			
13	6	0	94			
14		3	91			
15		6	88			
16		9	85			
17	8	0	92			
18		3	89			
19		6	86			
20		9	83			
21	10	0	90			
22		3	87			
23		6	84			
24		9	81			

**Table 2:** Summary of physical properties of the natural soil.

Property	Soil
Natural moisture content, %	16.11
Specific gravity, %	2.68
Liquid limit, %	61.7
Plastic limit, %	26.8
Plasticity index, %	34.9
Linear shrinkage, %	17.4
% passing sieve No. 200	60
Free swell, %	64.8
USCS classification	CH
Optimum moisture content, %	23
Maximum dry density, Mg/m^3^	1.63
Color	Reddish Brown
Dominant Soil Mineral	Kaolinite

**Table 3:** Summary of physical properties of the cement.

Property	Cement
Soundness, mm	0.38
Specific gravity, %	3.13
Fineness, m^2^/kg	362
3 days UCS, Mpa	29.1
Bulk density, kg/m^3^	1101
Initial Setting time, min	33.4
Final Setting time, min	242
Color	Ash

**Table 4:** Summary of physical properties of the sawdust ash.

Property	Sawdust Ash
Natural moisture content, %	26.07
Specific gravity, %	2.09
Liquid limit, %	22.5
Plastic limit, %	12.7
Plasticity index, %	10.2
Porosity, %	82.4
Fineness, micron	650
Rate of burning, g/hr	2224
Water absorption, %	18.23
Apparent	1.056
Linear shrinkage, %	5.05
Bulk density, kg/m^3^	412
Modulus of rupture, Mg/m^3^	1.249
Color	Ash

## Data Availability

Some or all data, models, or codes that support the findings of this study are available from the corresponding author upon reasonable request.

## Appendix A

**Table A1:** Two-way analysis of variance for specific gravity results of soil–cement–sawdust ash mixtures.

Property	Source of Variation	Degrees of Freedom	F_CAL_	*p*-Value	F_CRIT_	Remark
Specific gravity	Cement	3	66.22449	6.99 × 10^−09^	3.287382	F_CAL_ > F_CRIT_ Significant effect
	SDA	5	52.26531	5.85 × 10^−09^	2.901295	F_CAL_ > F_CRIT_ Significant effect

**Table A2:** Two-way analysis of variance for Atterberg limit results of soil–cement–sawdust ash mixtures.

Property	Source of Variation	Degrees of Freedom	F_CAL_	*p*-Value	F_CRIT_	Remark
Liquid limit	Cement	3	278.907	2.24 × 10^−13^	3.287382	F_CAL_ > F_CRIT_ Significant effect
	SDA	5	73.77099	5.08 × 10^−10^	2.901295	F_CAL_ > F_CRIT_ Significant effect
Plastic limit	Cement	3	25.74292	3.64 × 10^−06^	3.287382	F_CAL_ > F_CRIT_ Significant effect
	SDA	5	13.969	3.42 × 10^−05^	2.901295	F_CAL_ > F_CRIT_ Significant effect
Plasticity index	Cement	3	417.5243	1.14 × 10^−14^	3.287382	F_CAL_ > F_CRIT_ Significant effect
	SDA	5	47.25471	1.18 × 10^−08^	2.901295	F_CAL_ > F_CRIT_ Significant effect
Linear shrinkage	Cement	3	1289.157	2.58 × 10^−18^	3.287382	F_CAL_ > F_CRIT_ Significant effect
	SDA	5	47.10454	1.21 × 10^−08^	2.901295	F_CAL_ > F_CRIT_ Significant effect

**Table A3:** Two-way analysis of variance for compaction results of soil–cement–saw dust ash mixtures.

Property	Source of Variation	Degrees of Freedom	F_CAL_	*p*-Value	F_CRIT_	Remark
MDD	Cement	3	51.31148	4.04 × 10^−08^	3.287382	F_CAL_ > F_CRIT_ Significant effect
	SDA	5	20.90164	2.84 × 10^−06^	2.901295	F_CAL_ > F_CRIT_ Significant effect
OMC	Cement	3	39.33962	2.4 × 10^−07^	3.287382	F_CAL_ > F_CRIT_ Significant effect
	SDA	5	23.18868	1.45 × 10^−06^	2.901295	F_CAL_ > F_CRIT_ Significant effect

**Table A4:** Two-way analysis of variance for effect of compaction water on HC results of soil–cement–sawdust ash mixtures.

Property		Source of Variation	Degrees of Freedom	F_CAL_	*p*-Value	F_CRIT_	Remark
HC	−2%	Cement	3	17.47646	3.69 × 10^−05^	3.287382	F_CAL_ > F_CRIT_ Significant effect
		SDA	5	5.215878	5.68 × 10^−03^	2.901295	F_CAL_ > F_CRIT_ Significant effect
	0%	Cement	3	23.47024	6.43 × 10^−06^	3.287382	F_CAL_ > F_CRIT_ Significant effect
		SDA	5	6.148309	2.74 × 10^−03^	2.901295	F_CAL_ > F_CRIT_ Significant effect
	+2%	Cement	3	33.74422	6.53 × 10^−07^	3.287382	F_CAL_ > F_CRIT_ Significant effect
		SDA	5	12.55617	6.41 × 10^−05^	2.901295	F_CAL_ > F_CRIT_ Significant effect
	+4%	Cement	3	15.63979	6.93 × 10^−05^	3.287382	F_CAL_ > F_CRIT_ Significant effect
		SDA	5	4.894346	7.45 × 10^−03^	2.901295	F_CAL_ > F_CRIT_ Significant effect

**Table A5:** Two-way analysis of variance for HC results of soil–cement–sawdust ash mixtures.

Property	Source of Variation	Degrees of Freedom	F_CAL_	*p*-Value	F_CRIT_	Remark
HC	Cement	3	15.69096	6.8 × 10^−05^	3.287382	F_CAL_ > F_CRIT_ Significant effect
	SDA	5	4.788327	8.16 × 10^−03^	2.901295	F_CAL_ > F_CRIT_ Significant effect

**Table A6:** Two-way analysis of variance for effect of compaction water on VSS results of soil–cement–sawdust ash mixtures.

Property		Source of Variation	Degrees of Freedom	F_CAL_	*p*-Value	F_CRIT_	Remark
VSS	−2%	Cement	3	86.30367	1.67 × 10^−10^	3.287382	F_CAL_ > F_CRIT_ Significant effect
		SDA	5	112.3474	1.65 × 10^−10^	2.901295	F_CAL_ > F_CRIT_ Significant effect
	0%	Cement	3	155.1971	1.62 × 10^−11^	3.287382	F_CAL_ > F_CRIT_ Significant effect
		SDA	5	155.3852	2.3 × 10^−12^	2.901295	F_CAL_ > F_CRIT_ Significant effect
	+2%	Cement	3	22.14195	9.17 × 10^−06^	3.287382	F_CAL_ > F_CRIT_ Significant effect
		SDA	5	37.69041	5.65 × 10^−08^	2.901295	F_CAL_ > F_CRIT_ Significant effect
	+4%	Cement	3	27.30304	2.52 × 10^−06^	3.287382	F_CAL_ > F_CRIT_ Significant effect
		SDA	5	49.79083	8.22 × 10^−09^	2.901295	F_CAL_ > F_CRIT_ Significant effect

**Table A7:** Two-way analysis of variance for VSS results of soil–cement–sawdust ash mixtures.

Property	Source of Variation	Degrees of Freedom	F_CAL_	*p*-Value	F_CRIT_	Remark
VSS	Cement	3	18.64996	2.54 × 10^−05^	3.287382	F_CAL_ > F_CRIT_ Significant effect
	SDA	5	38.157	5.2 × 10^−08^	2.901295	F_CAL_ > F_CRIT_ Significant effect

**Table A8:** Two-way analysis of variance for effect of compaction water on UCS results of soil–cement–sawdust ash mixtures.

Property		Source of Variation	Degrees of Freedom	F_CAL_	*p*-Value	F_CRIT_	Remark
UCS	−2%	Cement	3	121.1904	9.72 × 10^−11^	3.287382	F_CAL_ > F_CRIT_ Significant effect
		SDA	5	68.19628	8.9 × 10^−10^	2.901295	F_CAL_ > F_CRIT_ Significant effect
	0%	Cement	3	56.60906	2.06 × 10^−08^	3.287382	F_CAL_ > F_CRIT_ Significant effect
		SDA	5	37.6517	5.69 × 10^−08^	2.901295	F_CAL_ > F_CRIT_ Significant effect
	+2%	Cement	3	59.14261	1.53 × 10^−08^	3.287382	F_CAL_ > F_CRIT_ Significant effect
		SDA	5	23.92532	1.19 × 10^−06^	2.901295	F_CAL_ > F_CRIT_ Significant effect
	+4%	Cement	3	56.24673	2.16 × 10^−08^	3.287382	F_CAL_ > F_CRIT_ Significant effect
		SDA	5	25.52665	7.75 × 10^−07^	2.901295	F_CAL_ > F_CRIT_ Significant effect

**Table A9:** Two-way analysis of variance for UCS results of soil–cement–sawdust ash mixtures.

Property	Source of Variation	Degrees of Freedom	F_CAL_	*p*-Value	F_CRIT_	Remark
UCS	Cement	3	56.24673	2.16 × 10^−08^	3.287382	F_CAL_ > F_CRIT_ Significant effect
	SDA	5	25.52665	7.75 × 10^−07^	2.901295	F_CAL_ > F_CRIT_ Significant effect
